# Sepsis in Latvia—Incidence, Outcomes, and Healthcare Utilization: A Retrospective, Observational Study

**DOI:** 10.3390/healthcare12020272

**Published:** 2024-01-21

**Authors:** Laura Puceta, Artis Luguzis, Uga Dumpis, Guna Dansone, Natalija Aleksandrova, Juris Barzdins

**Affiliations:** 1 Faculty of Medicine, University of Latvia, LV-1004 Riga, Latvia; 2 Department of Internal Medicine, Pauls Stradins Clinical University Hospital, LV-1002 Riga, Latvia; 3 Laboratory for Statistical Research and Data Analysis, Faculty of Physics, Mathematics and Optometry, University of Latvia, LV-1004 Riga, Latvia; 4 Department of Infectious Diseases and Hospital Epidemiology, Pauls Stradins Clinical University Hospital, LV-1002 Riga, Latvia

**Keywords:** sepsis, Latvia, healthcare utilization, incidence, outcomes, epidemiology, outpatient care, mortality rate, administrative data, healthcare systems

## Abstract

This study explores the incidence, outcomes, and healthcare resource utilization concerning sepsis in Latvia’s adult population. Using a merged database from the National Health Service and the Latvian Centre for Disease Prevention and Control, sepsis-related hospitalizations were analyzed from 2015–2020. Findings revealed a 53.1% surge in sepsis cases from 2015–2018 with subsequent stabilization. This spike was more prominent among elderly patients. The age/sex adjusted case fatality rate rose from 34.7% in 2015 to 40.5% in 2020. Of the 7764 sepsis survivors, the one-year mortality rate was 12% compared to 2.2% in a reference group of 20,686 patients with infections but no further signs of sepsis. Sepsis survivors also incurred higher healthcare costs, driven by longer rehospitalizations and increased pharmaceutical needs, though they accessed outpatient services less frequently than the reference group. These findings underscore the growing detection of sepsis in Latvia, with survivors facing poorer outcomes and suggesting the need for enhanced post-sepsis outpatient care.

## 1. Introduction

### 1.1. Sepsis: Evolving Definitions, Global Impact, and the Complexities of Epidemiological Assessments

Sepsis, defined as a life-threatening organ dysfunction caused by a dysregulated host response to infection [1], is a complex syndrome associated with long-term morbidity and major economic burden, affecting more than 30 million patients globally and responsible for estimated 11 million deaths per year worldwide [2,3]. A resolution from the World Health Organisation identified sepsis as a global health priority in 2017 [4,5]. True population-level sepsis incidence and mortality rates remain unknown; however, the estimates indicate that incidence is increasing. Several factors might account for this trend such as aging populations, increased awareness and detection due to heightened sepsis education in recent years, and variations in medical coding practices [6,7,8]. Defining sepsis has been challenging and there have been several revised definitions over the past decades with the latest addition of the so-called Sepsis-3 consensus document in 2016 [1].

About 50% of sepsis survivors encounter long-term sequelae, such as post-traumatic stress syndrome, anxiety, myalgias, repeated hospitalizations due to recurrent infection, worsening of chronic organ failure or acute conditions, e.g., myocardial infarction or stroke due to immunoparalysis and persistent inflammation. However, the association between long-term health trajectories and pre-existing comorbidities, age, and the clinical course of sepsis itself remains unclear [9,10].

Clinical data and administrative databases can be used for epidemiological assessments, though changes in sepsis definitions pose challenges to identify real-world incidence. A clinical chart review is a validated method to identify sepsis cases; however, it is not cost-effective for studying large cohorts. Another approach to sepsis surveillance is using a range of International Classification of Diseases Codes (ICD) in administrative databases. A notable limitation of this method is the code list variation in different studies, although the principle of the Angus implementation is commonly employed and ICD code lists consist of explicit codes for sepsis and implicit codes, which combine infection and organ dysfunction codes, thus corresponding to clinical sepsis criteria [11,12,13,14].

ICD case identification strategies were compared to the “golden standard” of manual patient chart review, and the studies have shown that sepsis coding in claims data has good specificity, but lacks sensitivity [13,14].

It is apparent that sepsis was considered as a “garbage code” in the Global Burden of Disease statistics for years, so sepsis-related deaths were classified as caused by the underlying infection. This practice complicates the comparison of study results over time since coding strategies have changed, at least partly due to changes in hospital reimbursement policy. Regardless of numerous attempts to address this issue in more recent studies, the true mortality of sepsis patients remains unknown [4].

### 1.2. Overview of the Latvian Healthcare System and Its Implications for Sepsis Research and Care

According to the National Central Statistical Bureau, in 2019, Latvia achieved a total life expectancy of 75.6 years, and the per capita gross domestic product at current prices reached EUR 15,980. Antimicrobial resistance proportions for 12 antibiotic bacterium pairs increased considerably between 2005 and 2019 (24.9% vs. 30.7%) and averaged above the EU/EEA average (21.3% in 2019) [15]. The country’s public healthcare system is primarily financed through government taxation. The National Health Service (NHS) operating under the Ministry of Health acts as the sole purchaser of health services. Services are predominantly provided by hospitals owned by the government or municipalities, whereas outpatient care institutions, to a larger extent than hospitals, are under private ownership. In comparison to other European Union countries, the public share of total health expenditure in Latvia is relatively modest, standing at just 61% in 2019 [16]. Privately purchased health services are covered through direct out-of-pocket payments or voluntary supplemental health insurance. This kind of insurance is increasingly becoming a part of employment benefit packages in Latvia and contributions by employers towards employee health insurance until certain limits are exempt from income tax and mandatory social insurance contributions [17]. By exempting voluntary health insurance from taxes and other contributions, the government indirectly acknowledges and addresses gaps in public healthcare funding.

Significant private spending as the distinctive characteristics of the Latvian healthcare system influences both the research to study sepsis through health administrative data and the care delivered to sepsis patients. In terms of research implications, it is important to note that while the NHS administrative data capture official, relatively minor personal co-payments for services it funds, other substantial private expenditures are not recorded. For instance, some patients, either due to financial capability or having voluntary insurance, may fully cover the cost of services to avoid long waiting times for NHS-sponsored services, opt for treatments without primary care referrals, or choose private providers. Such situations are commonly associated with outpatient services, including specialist consultations and diagnostics or elective procedures performed in both inpatient or outpatient settings. Additionally, there are out-of-pocket expenditures for services not under the NHS umbrella. This includes payments for pharmaceuticals which are not on the approved reimbursement list. Mentioned private spending pathways should be taken into account using NHS data for sepsis research. It also underscores the challenges sepsis survivors might face post-hospitalization. Specifically, while hospital treatments are usually accessible to all, those without sufficient funds or voluntary insurance might encounter barriers in obtaining adequate follow-up care.

### 1.3. Research Goals and Scope in Context of Global Sepsis Trends

Considering the widespread impact and complexities of sepsis on a global scale, this study is focused on gaining a thorough insight into the incidence, outcomes, and healthcare utilization of sepsis among Latvian adults. Taking into account the unique characteristics of Latvia’s healthcare system, we explore possibilities of identifying sepsis cases and subsequent care provided to survivors. We aim to draw conclusions that could be applied to various healthcare contexts worldwide.

## 2. Materials and Methods

### 2.1. Study Design and Objectives

This retrospective, observational study had two main objectives: firstly, to explore temporary trends regarding the incidence, patient characteristics, inpatient care outcomes, and costs of identifiable sepsis cases in Latvian hospitals in the study period from 2015 to 2020. Secondly, to assess the post-discharge care outcomes, healthcare utilization and costs among sepsis survivors and a comparable reference group within a year following their index hospitalization. Both objectives were explored using the same data source, employing identical procedures for extracting medico-administrative records, and relying on a uniform definition of sepsis case. While temporary trends analysis focuses on annual dynamics throughout the entire 6-year study period, the analysis of sepsis survivors includes patients discharged alive from 2015 to 2019 to ensure one-year follow-up time.

### 2.2. Data Source

Study data were sourced from a specialized research database (data link) maintained by the Latvian Centre for Disease Prevention and Control (CDPC) [18]. This database, consisting of anonymized NHS data linked to Causes of Death Registry, has been already tested in population-level studies in Latvia [19,20]. In line with the data access procedure, the CDPC, as the authorized agency, determines whether a study proposal is compliant with Latvian legislation. In this instance, approval by the Ethics Committee was considered unnecessary since only fully anonymized data were used.

Within the current study, access was granted to patients’ demographics (gender and age) and specific medico-administrative data, representing the provided inpatient and outpatient health care services as well as prescriptions for medicines fully or partially reimbursed by the NHS and dispensed at pharmacies. Regarding the hospital stay, details such as the service provider, principal and other set diagnoses, admission and discharge dates, discharge status codes (e.g., discharged to home, transferred, died), and costs covered by the NHS were available. For outpatient services, the data included the service provider, principal and other set diagnoses, start and end dates of the outpatient care episode, procedure codes, costs covered by the NHS, and the calculated co-payment charged to the patient. Pharmacy data included the prescription fill date, product codes, diagnosis, NHS reimbursement percentage (full, 75%, 50%), costs covered by the NHS, and costs charged to the patient. Additionally, for individuals who died in the study period, information from the Causes of Death Registry such as the date of death and the underlying cause of death was provided.

### 2.3. Study Population

The source population of the present study consists of adult individuals (age ≥ 18 years) who had at least one hospitalization with a diagnosis code indicating explicit sepsis or infection in any of the documented diagnoses. The International Statistical Classification of Diseases and Related Health Problems, Tenth Revision (ICD-10) codes were used to identify diagnoses. The applied case selection code list was adopted from prior research, particularly studies that employed secondary medico-administrative or cause-of-death data analysis. The explicit sepsis case was defined by the presence of at least one code from the list of explicit sepsis codes adapted from Pandolfi et al. [21]. The implicit sepsis case was defined by the presence of codes indicating both infection and organ dysfunction within the same hospitalization from the code list presented by Rudd et al. [3]. All diagnoses assigned to a hospitalization episode were used to define explicit and implicit sepsis cases.

The finalized version of code list for sepsis case identification (see Appendix A) included additional minor alteration, taking into account specifics of ICD-10 translation used in Latvian hospitals. For instance, R65.1 (defined as systemic inflammatory reaction with organ dysfunction of infectious origin/severe sepsis) was added to the explicit sepsis set, R57.2 (defined as septic shock) was moved from the implicit to the explicit code list, and R65.2 (defined as non-infectious origin of systemic inflammatory reaction without organ dysfunction) was excluded.

Subsequent hospitalizations for the same patient when the next admission date occurred on the same or next day after the previous discharge date were combined and considered as a single hospitalization episode for all analyses.

**Figure 1:**
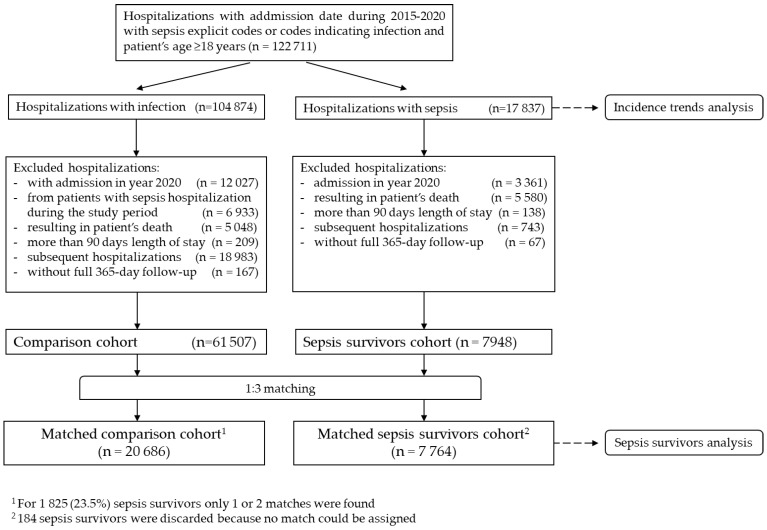
Creation of the study cohorts.

All sepsis hospitalizations (both implicit and explicit) from the source population were used in the analysis of sepsis incidence temporary trends. For the analysis of sepsis survivors, only the first hospitalization for each patient during the study period was used (further referred as the index hospitalization). All patients that did not have any sepsis hospitalization during the study period were used to form the reference cohort. In particular, these patients had at least one hospitalization with infection which did not meet the criteria for sepsis (as there was no documented organ dysfunction during the respective hospitalization), but no hospitalization with sepsis during the study period. The first hospitalization during the study period for each patient in the comparative group was considered. This cohort was used to study outcomes and post-discharge care for survivors. Further details regarding creation of sepsis and comparative cohorts are presented in Figure 1.

### 2.4. Baseline Patient Characteristics

For the identified sepsis hospitalization cases, patients’ age, gender, overall Charlson comorbidity index (CCI) [22,23], and presence of individual CCI comorbidity groups were considered. Comorbidities were ascertained based on respective ICD-10 codes in records from the 12-month period before the index hospitalization (inclusive). Records from all inpatient and outpatient health care episodes, as well as prescriptions for reimbursed medicines, were used, searching for relevant diagnostic codes. Furthermore, we categorized hospitalization cases based on the principal discharge diagnosis according to ICD-10 chapters.

### 2.5. Temporary Trends Analysis

To evaluate the temporary trends over the whole period, yearly data were analyzed separately, and all identified sepsis hospitalizations based on the criteria detailed in earlier sections were included (see Figure 1).

The summary of sepsis incidence was characterized by presenting numbers of sepsis hospitalizations, unique patients hospitalized, hospitalizations per patient and sepsis re-admissions, and sepsis incidence per 100,000 inhabitants (based on total number of hospitalizations). Mortality related to sepsis was characterized by total number of in-hospital deaths, case fatality rate (CFR), and deaths per 100,000 inhabitants. CFR was calculated as the number of sepsis hospitalizations ending with death divided by total number of sepsis hospitalizations.

Incidence and death per 100,000 inhabitants were age and sex standardized, considering 2015 as a reference year.

In addition, sepsis hospitalizations in each year were characterized by principal diagnosis at discharge, hospital length of stay (LOS), costs (expressed in EUR, representing case-based NHS payment to hospital as well as per-night calculated co-payment of EUR 10 charged to patient), and admitted patient characteristics, such as age, gender, and CCI comorbidities.

### 2.6. Analysis of Post-Discharge Outcomes and Care for Survivors

To evaluate the post-discharge outcomes and healthcare resource utilization in sepsis survivors as well as their baseline characteristics, sepsis patient and reference cohorts as described in Section 2.2 were used (see Figure 1). These outcomes included all-cause mortality, rehospitalization rates and durations, and the volume and cost of healthcare resources utilized. The follow-up period for both cohorts was one year after the index hospitalization, and the analysis was censored upon patient death.

In our analysis, health care utilization was captured in terms of both the quantity of services provided and their associated costs over the observation period. In calculation of costs of services provided to each patient, both payment from NHS budget as well as mandatory copayments from patients stipulated in respective regulation [24] and captured in a study data were accounted for. In the case of primary care financed by the NHS on capitation principle, the cost of the single visit to a general practitioner (GP) (EUR 17) was estimated by dividing the total budget for primary care (reported in NHS Annual reports [25]) by the total number of registered GP visits (reported by the NHS at the National Open Data Portal [26]).

The following outpatient care categories were distinguished: GP visits, specialist consultations, laboratory diagnostics, other outpatient care, and filled prescriptions of reimbursed medicines and medical products.

### 2.7. Statistical Analysis

In all descriptive analyses, categorical variables were summarized using counts and percentages, while continuous and count variables were summarized using medians and interquartile ranges (presented as first and third quartile).

In the analysis of sepsis survivors, a matched reference cohort was created from all candidate infection hospitalizations using propensity score matching. Propensity score was estimated using logistic regression with a binary sepsis indicator variable as outcome and baseline variables at discharge (age, sex, CCI score and individual comorbidities, LOS, hospitalization year) as covariates. A matching ratio of 1:3 was chosen to select a comparison cohort in order to obtain a reasonably large reference cohort which was also balanced sufficiently with respect to baseline characteristics between the comparative cohorts. In addition, to better account for temporal trends and the severity of hospitalization events, exact matching on the year of hospitalization and LOS was used. The quality of matching was assessed using standardized mean differences (SMDs) in variables included in the propensity score model after matching.

Descriptive analysis of defined outcomes was performed in each of the comparative cohorts as described above. In addition, *p*-values from Wilcoxon rank sum or Pearson’s Chi-squared test for continuous or categorical variables, respectively, were presented for comparison between the cohorts.

Kaplan–Meier (KM) survival curves were estimated for death and rehospitalization after discharge outcomes for each comparison cohort. The estimated risks for each outcome during the 1-year follow-up period were presented using the complement of the KM curve (1-KM).

## 3. Results

### 3.1. Results of Temporary Trends Analysis

#### 3.1.1. Sepsis Incidence Characteristics

In a six-year period from 2015 to 2020, a total of 17,837 admissions in Latvian hospitals were recorded (Table 1) with an implicit or explicit sepsis definition based on ICD-10 coding. Explicit sepsis cases constituted 43.4% of the total number of admissions. The majority of cases (approximately 96%) were the first admissions during the respective calendar year. The median number of admissions per patient is one, indicating that most patients had only one sepsis-related hospitalization. The re-admission rate (patients re-admitted within a year following their last admission) decreased from an average of 5.8% in 2015–2017 to 5.0% in 2018–2020.

There was a significant increase in detected sepsis cases, from 2177 to 3361, during the study period. However, this increase was not consistent throughout the whole six-year period. A pronounced 53.1% increase was noted in the first four years of the study period, with the annual number of cases subsequently leveling off. The total number of sepsis cases in the final three years, marked by stable dynamics, was 28.3% higher compared to the preceding three years.

The total age- and sex-standardized incidence of registered sepsis admissions experienced a 51.9% increase in the period from 2015 to 2018, reaching 166.5 cases per 100,000 population, and remained stable until the end of the study period.

#### 3.1.2. Mortality Characteristics

The non-adjusted case fatality rate increased from 34.7% in 2015 to 43.8% in 2020. However, the age- and sex-standardized case fatality rate increase was less prominent, at 34.7% and 40.5% in 2015 and 2020, respectively. An increase in age- and sex-standardized case fatality rate rises for the first three years of observation, but could be considered as stable for the rest of the period. The same pattern also characterizes the age- and sex-standardized death rate per 100,000 population.

#### 3.1.3. Hospitalization Characteristics

The median length of stay increased from 10 days in 2015 to 11 days in 2020. The calculated median cost for a hospital stay per case increased gradually during the study period, rising from EUR 691 in 2015 to EUR 1139 in 2020, reflecting a 94.7% growth.

#### 3.1.4. Patient Characteristics

The gender distribution shows that males accounted for slightly less than half of the cases, with a similar distribution over time.

The median age of sepsis patients increased slightly over the years, with the majority of cases occurring in the 60–79 age group. Notably, the increase in the number of registered cases was predominantly due to a rise in sepsis cases detected among older patients; the rise for those aged below 40 was a mere 3.4%, while being 17.9% in the 40–59 group, 20.3% in the 60–79 group, and reaching 33.3% for patients 80 and older.

Concomitant with the absolute and relative increase in patients in older age groups, the overall characteristics of sepsis patients were also altered. Comparing the first and the last year of the study period, the Charlson comorbidity index increased from 2 (IQR 1, 4) to 3 (IQR 1, 4). Among the most common comorbidities were congestive heart failure, increasing from 17.0% to 52.8%, and cerebrovascular disease, increasing from 21.5% to 37.2%, while chronic pulmonary disease decreased from 24.5% to 21.7%. Notably, there was also a gradual increase in the prevalence of conditions such as dementia and moderate-to-severe renal disease over the study period.

Diseases of the respiratory system were the leading principal diagnosis upon discharge. In general, the distribution of the principal diagnosis codes to particular ICD-10 chapters did not demonstrate substantial change over the period, with the exception of the year 2020, when 435 (4.7%) of hospitalization cases attributable to sepsis were for the first time coded with a new special purpose code introduced for COVID-19 patients. Correspondingly, in 2020, there was a drop in principal discharge diagnoses coded as ‘Diseases of the Respiratory System’ or ‘Certain Infectious and Parasitic Diseases’.

### 3.2. Post-Discharge Outcomes and Health Care Utilization in Sepsis Survivors

A total of 7764 unique patients who were discharged alive following their first hospitalization for sepsis between 2015 and 2019 were identified and matched with comparators. They were compared to a reference cohort comprising 20,686 patients who were hospitalized and discharged alive during the same period with diagnostic codes indicative of infection, but did not meet the criteria for sepsis (Table 2).

In general, a good balance in baseline characteristics was achieved between the two comparison cohorts after matching. SMDs between the cohorts were larger than 0.1 only, first, for LOS, due to slightly longer hospitalizations in the sepsis cohort, and, second, for CCI score, driven by differences in moderate-to-severe renal and moderate-to-severe liver disease prevalence between the cohorts.

**Figure 2:**
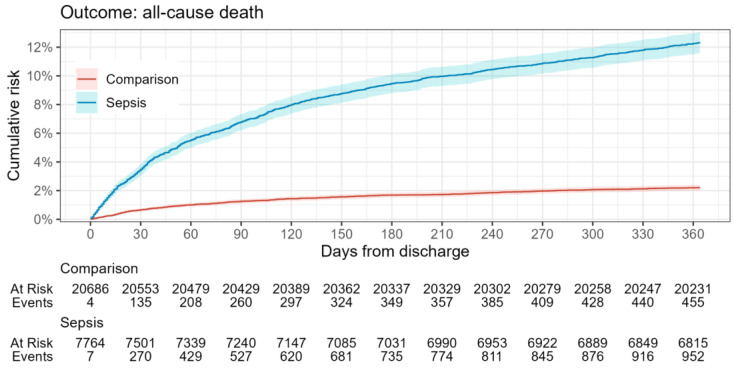
All-cause mortality risk in sepsis survivors during the one-year follow-up period.

**Figure 3:**
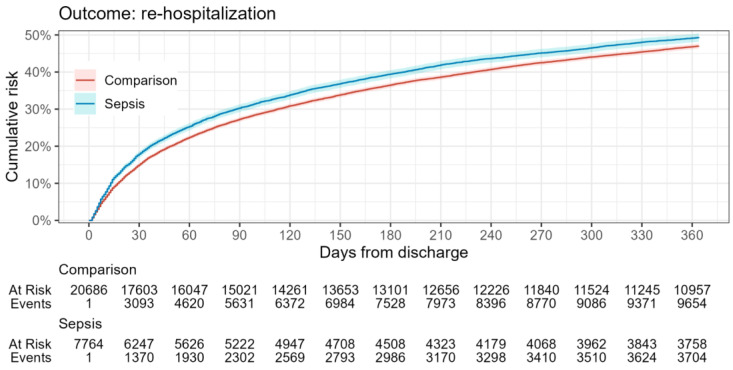
Rehospitalization risk in sepsis survivors during the one-year follow-up period.

In relation to the defined outcomes (Table 3, Figure 2 and Figure 3), analysis shows that the mortality rate within 365 days was significantly higher in the sepsis group relative to the reference group (12% vs. 2%), while the rehospitalization rate between groups did not differ significantly. This highlights the impact of sepsis on overall survival probability.

Both groups demonstrate substantial healthcare utilization, with notably high rehospitalization rates, consistent across both groups. This indicates that patients from each group required intensive medical follow-up. Sepsis survivors experienced a longer duration of hospital stays, higher rehospitalization costs, and an increased cost of filled pharmaceutical prescriptions relative to the reference group. Despite these findings, sepsis survivors sought outpatient care less frequently. This includes fewer visits to primary care physicians, fewer specialist consultations, and less other outpatient care with the exception of laboratory diagnostics. Moreover, the overall outpatient care costs were also significantly lower for the sepsis cohort compared to the reference group.

## 4. Discussion

In this first longitudinal, population-based study of sepsis in Latvia, we found a significant gradual increase in the incidence of identified sepsis cases among hospitalized patients during the first half of observational period, followed by relative stability in the second half of the study when the incidence rate reached 166 cases per 100,000 population. Several recent studies focusing on sepsis have also reported a rise in incidence. For instance, the study by Dupuis et al. [27] and the research of Fleischmann-Struzek et al. [7], both observing the period from 2010 to 2015, reported an increase in sepsis incidence; Dupuis et al. observed an increase from 135 to 171 cases of sepsis and septic shock per 100,000 population in France, while Fleischmann-Struzek et al. noted an incidence rise from 108 to 158 in explicit sepsis cases per 100,000 population in Germany. The most recent study by Pandolfi et al. [21], covering the period from 2015 to 2019, reveals an increase in bacterial sepsis incidence in France from 357 to 403 per 100,000 population. Within the horizon of the present study, we are unable to conclude whether the higher incidence of sepsis cases among patients reflects a genuine increase in sepsis incidence or rather an improvement in sepsis detection, particularly among elderly patients, given that the observed rise in cases was not evident in younger age groups. However, the fact that the increase in sepsis cases leveled off in the last three years of our study suggests that the earlier rise probably was not only due to factors like an aging population and more people having chronic health conditions. Instead, it seems to be at least partially linked to better recognition of sepsis patients and improved coding practices. Interestingly, in a previous study by Rhee et.al. [28] the authors question the sepsis incidence increase detected in administrative data using ICD codes, since the trend was not matched by comparable increase in ICD-documented infection rates. However, we did not analyze infection rates in this study. Challenges in sepsis recognition are extensively discussed among researchers in the field, and underestimation of sepsis cases in administrative data was observed, for instance, by up to 3.5-fold by explicit coding in a single center validation study in Germany [29]. The undercoding of sepsis cases was also emphasized in other studies, especially for explicit cases which constituted a substantial part of the cases in our study as well; in particular, these studies found that less than 50% of sepsis patients with organ dysfunction in the United States and 15% in Sweden received an explicit ICD code for sepsis [30,31]. Therefore, better sepsis recognition and improved coding practices are plausible causes for increasing sepsis incidence rates. To our knowledge, there have not been official sepsis awareness-raising campaigns among healthcare providers during the study period; however, sepsis may have been discussed more frequently in the context of publishing the Sepsis-3 consensus document in 2016.

When comparing Latvia’s observed sepsis incidence rate with most recent findings from other studies, discrepancies suggest that sepsis may still not be adequately recognized in Latvian hospitals. For example, per 100,000 population, Yébenes et al. [32] recorded 212.7 cases in Catalonia in 2017, Kim et al. [33] found 395.1 cases in Korea in 2019, Pandolfi et al. [21] noted 403 cases in France in 2022, and Li et al. documented 1162.8 cases in Australia in 2021 [34]. At the same time, it must be acknowledged that variations in the methodology and sepsis definition used for sepsis case detection can lead to these disparities in reported incidence rates. For example, in contrast to the Pandolfi study, we focused on the identification of organ dysfunction solely through ICD-10 codes, without delving into additional data that might indicate organ support interventions or intensive care unit (ICU) stays. It is worth noting that our study specifically targeted the adult population, while some other studies also covered pediatric groups. However, we believe that these methodological nuances alone cannot explain the vast difference in estimated incidence rates.

Unlike other studies that report a stabilization [33] or even a gradual decline [7,21,32,35], in the proportion of sepsis hospitalizations ending with patient death, Latvia has seen a significant increase in both the in-case fatality rate and in-hospital mortality per 100,000 population throughout the observation period. This suggests that previously mentioned increase in sepsis incidence is not attributable to better recognition of milder cases and a coding artifact known as “upcoding”, which would have resulted in decreasing mortality as pointed out in reports from Rhee and Klompas [8] and Fleischmann-Struzek et al. [36]. However, as the overall incidence of sepsis stabilized in the second half of the observation period, the surge in hospital mortality became less pronounced. However, the observed 40.5% case fatality rate is high compared to other studies on overall sepsis hospitalization outcomes: 21.6% reported by Yébenes et al. [32], 23.6% reported by Pandolfi et al. [21], and 27% reported by Fleischmann-Struzek et al. [37]. It is comparable to the 41.7% reported by Fleischmann-Struzek et al. [7] for patients with explicitly coded severe sepsis. The case fatality rate in this study was lower than reported mortality of 51% from one of the Latvian university hospitals where patients with sepsis-related ICD-10 codes upon discharge were enrolled; however, cases were validated by a chart review [38].

This high case fatality rate might suggest potential inadequacies in hospital care for sepsis in Latvia which could be influenced by the financial framework within which these hospitals operate. The NHS calculated costs reflected in the analyzed data correspond to the payments made to hospitals based on predefined tariffs, rather than the actual expenses incurred for individual cases. This discrepancy was highlighted in a 2016 study analyzing severe sepsis manifestations and economic dimensions in an intensive care unit in Latvia [39], which revealed an average cost for one patient to be EUR 2226, significantly higher than median payment of EUR 1139 reimbursed by the NHS in 2020. When compared to costs reported in countries like France, where median costs for hospitalization episodes were EUR 11,400 for patients with sepsis and EUR 16,439 for patients with septic shock [27], the financial constraints become clearly evident. Even after adjusting for purchasing power parity, the pronounced disparities in funding and treatment resources are likely to influence sepsis patient outcomes. Further research into hospital care quality and its relationship with funding is essential to understand these observations and their potential implications fully.

In contrast to the high case fatality rate in Latvian hospitals, the observed 12% one-year mortality rate for sepsis survivors is relatively low. For comparison, studies focusing on one-year mortality rates for patients discharged after an initial sepsis hospitalization report figures such as 12.5% [40], 15% [41], 22.3% [42], 23% [43], and even as high as 30.7% [37,44]. Furthermore, a meta-analysis indicates a post-acute sepsis mortality rate of 16.1% at one year, derived by estimating the difference between cumulative one-year mortality and acute mortality across 43 studies that did not specifically focus on this outcome [45]. The discrepancy between a high in-hospital mortality rate for sepsis patients in Latvia and the relatively low one-year mortality rate for sepsis survivors post-discharge presents an intriguing paradox. One plausible explanation is that the patients being admitted to hospitals in Latvia are generally afflicted with a more severe course of disease. This would result in a high in-hospital mortality rate, but the patients who do survive and are discharged may represent a subset with a relatively better prognosis.

This notion is indirectly supported by the observed 48% rehospitalization rate for sepsis survivors in Latvia during the first-year post-discharge. This rate is lower than the 73.8% reported by Pandolfi, Brun-Buisson, et al. for France [42], suggesting a potentially better health status for sepsis survivors in Latvia. However, when comparing the rehospitalization rate in Latvia to the average 39.0% one-year rate highlighted in a meta-analysis by Shankar-Hari et al. [46], this interpretation becomes less straightforward.

While the rehospitalization rates provide crucial insights into post-sepsis patient care, another dimension deserving attention is the pattern of ambulatory healthcare utilization among sepsis survivors, but in our investigation of this aspect we encountered limited referential benchmarks in the current literature. To the best of our knowledge, ours is the first study estimating outpatient services utilization of sepsis survivors using nationwide data. This contrasts the broad consensus that a structured post-sepsis care regimen is needed to improve long term outcomes of sepsis survivors due to sepsis-induced health consequences [5,9].

A study by Schmidt et al. [47] provides some comparative insights. By tracking 210 sepsis patients after discharge from ICU in Germany, they determined an average one-year healthcare expense of EUR 26,559—of which EUR 19,542 accounted for rehospitalizations, leaving EUR 7017 for outpatient visits, rehabilitation, and pharmaceuticals. These figures suggest a significantly more intense outpatient care for sepsis survivors in Germany compared to Latvia. However, several nuances complicate direct comparison: specifically, the disparities in purchasing power between the countries, the specific focus of the German study on ICU-treated sepsis patients (a criterion not mandated in ours), and the notable dependence of Latvian ambulatory healthcare on private funding and out-of-pocket expenditures which is not represented in our dataset.

In light of these complexities our methodological approach—comparing sepsis survivors to patients discharged following hospitalizations for infections but not diagnosed with sepsis—provides more robust evidence of apparent care gaps for sepsis survivors. They have fewer primary care visits to general practitioners, fewer specialist consultations, and less frequent non-laboratory diagnostic interventions. Additionally, the sepsis cohort incurred significantly lower expenses for outpatient care in comparison to the reference group. This reduced outpatient care may contribute to both the notably elevated mortality rates that sepsis survivors face in the year post-discharge and to their more prolonged and costlier rehospitalizations compared to the non-sepsis patient group.

One of the strengths of our research is that it is the first longitudinal, population-based exploration of sepsis in Latvia, making use of a comprehensive database from authoritative health institutions spanning from 2015 to 2020. This timeframe offers a thorough view of sepsis trends and outcomes in the region. Additionally, our innovative comparative approach, contrasting sepsis survivors against a reference group of infection patients without sepsis, is particularly enlightening regarding post-sepsis care disparities.

However, there are also limitations. In the scope of our study, we cannot determine whether the observed increase in sepsis cases results from a genuine rise in incidence or improved detection techniques. In addition, our study might not capture the full spectrum of post-sepsis ambulatory healthcare utilization due to substantial out-of-pocket expenses in Latvia’s outpatient care system. Lastly, we acknowledge the absence of data on causative microorganisms, multidrug resistance, and specific severity metrics, like organ support interventions or ICU stays, which might influence the comparability of our findings with other global studies.

## 5. Conclusions

In conclusion, our study underscores the importance of comprehensive care for sepsis survivors in Latvia, revealing notable inconsistencies in post-hospitalization outpatient services. Our findings not only inform strategies within Latvia’s healthcare system, but also offer insights that could be valuable in broader global contexts.

## Figures and Tables

**Table 1:** Temporary trends in sepsis hospitalizations 2015–2020.

Characteristic	2015	2016	2017	2018	2019	2020	Trend 2015–2020 ^1^
**Sepsis incidence characteristics:**							
Number of hospitalizations	2177	2780	2855	3334	3330	3361	↗
Number of unique patients	2090 (96.0%) ^2^	2672 (96.1%)	2746 (96.2%)	3188 (95.6%)	3199 (96.1%)	3233 (96.2%)	↗
Number of admissions, median (IQR)	1 (1, 1)	1 (1, 1)	1 (1, 1)	1 (1, 1)	1 (1, 1)	1 (1, 1)	NS
Re-admissions (during the year following the last admission)	131 (6.0%)	142 (5.1%)	177 (6.2%)	203 (6.1%)	180 (5.4%)	N/A	NS
Incidence per 100,000 (age/sex standardized)	109.6	139.8	143.3	166.5	165.6	165.2	↗
**Mortality characteristics:**							
In-hospital death cases	756 (34.7%)	1036 (37.3%)	1045 (36.6%)	1387 (41.6%)	1356 (40.7%)	1472 (43.8%)	↗
Case fatality (age/sex standardized)	34.7%	36.5%	35.1%	39.9%	38.6%	40.5%	↗
Deaths per 100,000 (age/sex standardized)	38.1	51.9	51.9	68.3	65.8	70.5	↗
**Hospitalization characteristics:**							
Hospital LOS (days), median (IQR)	10 (5, 18)	10 (5, 19)	10 (5, 19)	10 (5, 19)	11 (5, 20)	11 (6, 20)	↗
Cost (EUR), median (IQR)	691 (349, 1460)	678 (345, 1415)	679 (354, 1310)	822 (424, 1565)	973 (511, 1876)	1139 (628, 2042)	↗
**Patient characteristics:**							
Male sex	1016 (47%)	1300 (47%)	1334 (47%)	1541 (46%)	1577 (47%)	1579 (47%)	↗
Age, years median (IQR)	68 (52, 79)	69 (55, 79)	71 (56, 81)	71 (57, 81)	71 (56, 81)	72 (59, 81)	↗
Age 19–39	308 (15%)	343 (12%)	334 (11%)	361 (11%)	373 (11%)	286 (9%)	NS
Age 40–59	448 (21%)	507 (18%)	498 (17%)	605 (18%)	598 (18%)	567 (17%)	↗
Age 60–79	902 (41%)	1 236 (44%)	1 226 (43%)	1392 (42%)	1377 (41%)	1451 (43%)	↗
Age > 80	519 (24%)	694 (25%)	797 (28%)	976 (29%)	982 (29%)	1057 (31%)	↗
Charlson comorbidity score, median (IQR)	2 (1, 4)	2 (1, 4)	2 (1, 4)	3 (1, 4)	2 (1, 4)	3 (1, 4)	NS
**Comorbidities quantity (Charlson definitions):**							
Prior myocardial infarction	299 (14%)	394 (14%)	431 (15%)	464 (14%)	509 (15%)	457 (14%)	↗
Congestive heart failure	1036 (48%)	1440 (52%)	1505 (53%)	1771 (53%)	1731 (52%)	1790 (53%)	↗
Peripheral vascular disease	361 (17%)	554 (20%)	572 (20%)	653 (20%)	682 (20%)	729 (22%)	↗
Cerebrovascular disease	650 (30%)	926 (33%)	1002 (35%)	1194 (36%)	1237 (37%)	1301 (39%)	↗
Dementia	109 (5%)	155 (6%)	176 (6%)	227 (7%)	258 (8%)	288 (9%)	↗
Chronic pulmonary disease	518 (24%)	663 (24%)	732 (26%)	809 (24%)	723 (22%)	648 (19%)	NS
Rheumatologic disease	54 (3%)	57 (2%)	72 (3%)	84 (3%)	77 (2%)	76 (2%)	↗
Peptic ulcer disease	106 (5%)	116 (4%)	149 (5%)	159 (5%)	143 (4%)	127 (4%)	NS
Mild liver disease	164 (8%)	189 (7%)	204 (7%)	234 (7%)	197 (6%)	232 (7%)	NS
Diabetes	123 (6%)	183 (7%)	241 (8%)	259 (8%)	266 (8%)	311 (9%)	↗
Diabetes with chronic complications	229 (11%)	340 (12%)	329 (12%)	410 (12%)	464 (14%)	500 (15%)	↗
Cerebrovascular (hemiplegia) event	34 (2%)	33 (1%)	27 (1%)	50 (2%)	53 (2%)	56 (2%)	↗
Moderate-to-severe renal disease	335 (15%)	522 (19%)	601 (21%)	637 (19%)	747 (22%)	752 (22%)	↗
Cancer without metastases	268 (12%)	360 (13%)	379 (13%)	444 (13%)	429 (13%)	448 (13%)	↗
Moderate or severe liver disease	188 (9%)	270 (10%)	217 (8%)	253 (8%)	236 (7%)	237 (7%)	NS
Metastatic solid tumor	86 (4%)	64 (2%)	81 (3%)	91 (3%)	95 (3%)	106 (3%)	NS
Acquired immune deficiency syndrome	42 (2%)	51 (2%)	43 (2%)	64 (2%)	49 (2%)	55 (2%)	NS
**Principal diagnosis at discharge (ICD-10 chapters):**							
Diseases of the respiratory system (J00-J99)	583 (27%)	733 (26%)	750 (26%)	859 (26%)	778 (23%)	538 (16%)	NS
Certain infectious and parasitic diseases (A00-B99)	373 (17%)	443 (16%)	453 (16%)	516 (15%)	565 (17%)	452 (13%)	NS
Diseases of the circulatory system (I00-I99)	281 (13%)	417 (15%)	407 (14%)	514 (15%)	570 (17%)	482 (14%)	↗
Diseases of the genitourinary system (N00-N99)	181 (8%)	284 (10%)	312 (11%)	353 (11%)	356 (11%)	386 (11%)	↗
Diseases of the digestive system (K00-K93)	163 (8%)	201 (7%)	191 (7%)	287 (9%)	229 (7%)	264 (8%)	NS
Neoplasms (C00-D48)	200 (9%)	188 (7%)	220 (8%)	214 (6%)	177 (5%)	216 (6%)	NS
Pregnancy, childbirth, and the puerperium (O00-O99)	158 (7%)	199 (7%)	174 (6%)	194 (6%)	207 (6%)	147 (4%)	NS
Injury, poisoning, and certain other consequences of external causes (S00-T98)	59 (2.7%)	76 (2.7%)	94 (3.3%)	109 (3.3%)	104 (3.1%)	111 (3.3%)	↗
Codes for special purposes (U00-U85)	0 (0%)	0 (0%)	0 (0%)	0 (0%)	0 (0%)	473 (14%)	NS
Others	179 (8%)	239 (9%)	254 (9%)	288 (9%)	343 (10%)	292 (9%)	↗

^1^ Test of trend or linear regression for values: ↗ a significant increase is observed with a *p* value < 0.05; ‘NS’ no significant trend is observed; ^2^ Percentages are calculated with respect to number of admissions.

**Table 2:** Sepsis and reference cohort characteristics.

Characteristic	Sepsis Cohort N = 7764 ^1^	Reference Cohort N = 20,686 ^1^	SMD ^2^
Age (years)	67 (49, 78)	67 (48, 79)	0.01
Gender (male)	3343 (43%)	8281 (40%)	0.06
Length of stay of index hospitalization (days)	11 (7, 19)	10 (7, 16)	0.2
CCI score, median	2 (0, 3)	2 (0, 3)	0.14
**Comorbidities (Charlson definitions)**			
Prior myocardial infarction	919 (12%)	2290 (11%)	0.02
Congestive heart failure	3485 (45%)	9048 (44%)	0.02
Peripheral vascular disease	1019 (13%)	2508 (12%)	0.03
Cerebrovascular disease	2162 (28%)	5629 (27%)	0.01
Dementia	313 (4%)	741 (4%)	0.02
Chronic pulmonary disease	1913 (25%)	4915 (24%)	0.02
Rheumatologic disease	156 (2%)	393 (2%)	0.01
Peptic ulcer disease	305 (4%)	700 (3%)	0.03
Mild liver disease	537 (7%)	1345 (7%)	0.02
Diabetes	522 (7%)	1319 (6%)	0.01
Diabetes with chronic complications	870 (11%)	2045 (10%)	0.04
Cerebrovascular (hemiplegia) event	77 (1%)	165 (1%)	0.02
Moderate to severe renal disease	1313 (17%)	2729 (13%)	0.10
Cancer without metastases	896 (12%)	2248 (11%)	0.02
Moderate to severe liver disease	369 (5%)	205 (1%)	0.23
Metastatic solid tumor	141 (2%)	369 (2%)	0.00
Acquired immune-deficiency syndrome	116 (2%)	255 (1%)	0.02
**Year of discharge for index hospitalization**			
2015	1265 (16%)	3633 (18%)	0.04
2016	1558 (20%)	4201 (20%)	
2017	1610 (21%)	4386 (21%)	
2018	1643 (21%)	4248 (21%)	
2019	1687 (22%)	4207 (20%)	

^1^ Median (IQR); n (%), ^2^ Standardized mean difference.

**Table 3:** Sepsis survivors 2015–2019 post-discharge outcomes and health care use.

Characteristic	Sepsis Cohort N = 7764 ^1^	Reference Cohort N = 20,686 ^1^	*p*-Value ^2^
Death in 365 days	957 (12%)	458 (2%)	<0.001
Rehospitalization in 365 days	3721 (48%)	9697 (47%)	0.110
**Healthcare utilization volumes (per patient)**			
Inpatient care:			
Number of rehospitalizations per patient	0 (0, 1)	0 (0, 1)	0.042 *
Length of stay for all rehospitalizations (days)	13 (6, 27)	12 (5, 23)	<0.001
Outpatient care:			
Number of outpatient care episodes total	8 (3, 17)	9 (3, 18)	0.002
Primary care physician visits	2 (1, 5)	3 (1, 6)	0.015
Specialist consultations	1 (0, 3)	1 (0, 4)	<0.001
Laboratory diagnostics	2 (0, 5)	2 (0, 5)	0.3
Other outpatient care	1 (0, 3)	1 (0, 3)	<0.001 **
Pharmaceuticals prescriptions			
Number of filled prescriptions	5 (0, 17)	5 (0, 16)	<0.001
**Healthcare utilization costs (per patient EUR) ^3^**			
Inpatient care (rehospitalizations)	1027 (457, 2 359)	920 (438, 1 999)	<0.001
Outpatient care (total)	183 (73, 415)	211 (87, 447)	<0.001
Primary care physician visits	74 (37, 130)	74 (37, 130)	0.3
Specialist consultations	47 (21, 114)	56 (22, 136)	<0.001
Laboratory diagnostics	34 (15, 72)	33 (16, 67)	0.2
Other outpatient care	65 (19, 2 018)	73 (23, 222)	0.005
Pharmaceuticals	210 (66, 609)	175 (60, 477)	<0.001

^1^ Median (IQR); n (%), ^2^ Wilcoxon rank sum test; Pearson’s Chi-squared test, ^3^ Patients with at least one healthcare utilization episode, * in favor of the sepsis cohort, ** in favor of the reference cohort.

## Data Availability

Data are contained within the article.

## Appendix A. ICD-10 Codes Used for Selection of Patients

**Explicit Sepsis Codes**	**Infection Codes**	**Codes for Organ Dysfunction**
A02.1, A20.7, A22.7, A26.7, A32.7, A40-A40.9, A41-A41.9, A54.8, B00.7, B37.7, B44.7, B45.7, B46.4, B50.8, O85, O88.3, R57.2, R57.8, R651	A01-A02.0, A02.2, A02.9, A02.8, A03-A09.9, A19-A20.3, A20.8, A20.9, A21.7, A21.8, A21.9, A21-A21.3, A22.8, A22.9, A22-A22.2, A23-A24.0, A241-A44, A24.9, A25-A26.0, A26.8, A26.9, A27-A28.1, A28.2, A28.8, A28.9, A31-A32.12, A32.8, A32.9, A36-A39, A39.1-A39.3, A39.0, A39.4, A39.5, A39.8, A39.9, A42.7, A42.8, A42.9, A42-A42.2, A43-A46.0, A48.0-A49.9, A50-A50.9, A59-A59.9, A65-A65.0, A69-A69.1, A74, A74.8-A75.9, A77-A81.9, A83-A96.9, A98-B00.59, B00.8, B00.9, B01-B10.89, B25-B27.99, B29.4, B33-B34.9, B37-B37.6, B37.8, B37.9, B38-B50.9, B54-B55, B55.1-B55.9, B58-B60.8, B64, B67-B67.99, B91, B95-B99.9, G00-G08.0, G14-G14.6, H05.01-H05.039, H60.2-H60.23, H70.0-H70.009, I00, I02, I02.9, I26.01-I26.09, I26.90-I26.99, I33-I33.9, I38-I39.9, I40.0-I40.9, I76, I96-I96.9, I98.1, J01-J06.9, J09-J22.9, J36-J36.0, J39.0-J39.1, J85-J86.9, K35-K37.9, K57-K57.93, K61-K61.4, K63.0-K63.1, K65-K65.9, K67.8, K75.0-K75.1, K75.3, K76.3, K77.0, K81.0, K81.2, K83.0, K95.01, K95.81, L02-L08.9, M00-M02.9, M86-M86.9, M89.6-M89.69, N10-N10.9, N15.1-N15.9, N30-N30.91, N39.0, N41.0, N41.2-N41.3, N45-N45.9, N70-N77.8, N98.0, O03.3, O03.5, O03.8, O04.5, O04.8, O07.3, O08.0, O08.8, O23-O23.9, O03.0, O41.1, O41.8-O41.9, O75.3, O86-O86.8, O91, O91.0, O91.1, O91.2, O98-O98.9, R65.9, R65.0, R65.9, R78.81, T80.2-T80.29, T81.4, T82.6-T82.7, T83.5, T83.6, T84.5-T84.7, T85.7, T88.0, U04	D65, D68.9, D695, D69.6, D69.8, D69.9, E87.2-E87.8, G93.4, I46.0-I46.9, I95.1-I95.9, J80, J95.2, J95.3, J96.0-J96.9, K72.0-K72.9, N00-N00.9, N01-N01.9, N16.0, N17.0-N17.9, R09.2, R40.0-R40.2, R39.2, R41.8, R55, R57, R57.0, R57.1, R57.9

## References

[1] Singer M., Deutschman C.S., Seymour C.W., Shankar-Hari M., Annane D., Bauer M., Bellomo R., Bernard G.R., Chiche J.-D., Coopersmith C.M. (2016). The Third International Consensus Definitions for Sepsis and Septic Shock (Sepsis-3). JAMA.

[2] Fleischmann M.C., Scherag A., Adhikari N.K.J., Hartog C.S., Tsaganos T., Schlattmann P., Angus D.C., Reinhart K., International Forum of Acute Care Trialists (2016). Assessment of Global Incidence and Mortality of Hospital-treated Sepsis. Current Estimates and Limitations. Am. J. Respir. Crit. Care Med..

[3] Rudd K.E., Johnson S.C., Agesa K.M., Shackelford K.A., Tsoi D., Kievlan D.R., Colombara D.V., Ikuta K.S., Kissoon N., Finfer S. (2020). Global, regional, and national sepsis incidence and mortality, 1990–2017: Analysis for the Global Burden of Disease Study. Lancet.

[4] Reinhart K., Daniels R., Kissoon N., Machado F.R., Schachter R.D., Finfer S. (2017). Recognizing Sepsis as a Global Health Priority—A WHO Resolution. New Engl. J. Med..

[5] World Health Organisation Executive Board (EB140/12) Improving the Prevention, Diagnosis and Clinical Management of Sepsis. 9 January 2017. https://apps.who.int/gb/ebwha/pdf_files/EB140/B140_12-en.pdf.

[6] Klompas M., Rhee C. (2016). Sepsis and the theory of relativity: Measuring a moving target with a moving measuring stick. Crit. Care.

[7] Fleischmann-Struzek C., Mikolajetz A., Schwarzkopf D., Cohen J., Hartog C.S., Pletz M., Gastmeier P., Reinhart K. (2018). Challenges in assessing the burden of sepsis and understanding the inequalities of sepsis outcomes between National Health Systems: Secular trends in sepsis and infection incidence and mortality in Germany. Intensiv. Care Med..

[8] Rhee C., Klompas M. (2020). Sepsis trends: Increasing incidence and decreasing mortality, or changing denominator?. J. Thorac. Dis..

[9] Prescott H.C., Angus D.C. (2018). Enhancing Recovery from Sepsis: A Review. JAMA.

[10] Prescott H.C., Iwashyna T.J., Blackwood B., Calandra T., Chlan L.L., Choong K., Connolly B., Dark P., Ferrucci L., Finfer S. (2019). Understanding and Enhancing Sepsis Survivorship. Priorities for Research and Practice. Am. J. Respir. Crit. Care Med..

[11] Angus D.C., Linde-Zwirble W.T., Lidicker J., Clermont G., Carcillo J., Pinsky M.R. (2001). Epidemiology of severe sepsis in the United States: Analysis of incidence, outcome, and associated costs of care. Crit. Care Med..

[12] Martin G.S., Mannino D.M., Eaton S., Moss M. (2003). The Epidemiology of Sepsis in the United States from 1979 through 2000. N. Engl. J. Med..

[13] Iwashyna T.J., Odden A., Rohde J., Bonham C., Kuhn L., Malani P., Chen L., Flanders S. (2014). Identifying Patients with Severe Sepsis Using Administrative Claims: Patient-Level Validation of the Angus Implementation of the International Consensus Conference Definition of Severe Sepsis. Med. Care.

[14] Jolley R.J., Quan H., Jetté N., Sawka K.J., Diep L., Goliath J., Roberts D.J., Yipp B.G., Doig C.J. (2015). Validation and optimisation of an ICD-10-coded case definition for sepsis using administrative health data. BMJ Open.

[15] OECD (2023). Embracing a One Health Framework to Fight Antimicrobial Resistance. OECD Health Policy Studies.

[16] (2021). OECD/European Observatory on Health Systems and Policies, Latvia: Country Health Profile 2021, State of Health in the EU. OECD Publishing: Paris, France. https://eurohealthobservatory.who.int/publications/m/latvia-country-health-profile-2021.

[17] Briģis Ģ., Sagan A., Thomson S. (2016). Latvia. Voluntary Health Insurance in Europe: Country Experience.

[18] SPKC Dati Pētniecībai no Monitorēšanas Sistēmas|Slimību Profilakses un Kontroles Centrs [In Latvian]. https://www.spkc.gov.lv/lv/dati-petniecibai-no-monitoresanas-sistemas.

[19] Barzdins J., Luguzis A., Valeinis J., Lepiksone J., Skrule J., Pildava S., Konstante R. (2021). Towards evidence-based management: A nationwide administrative data-based audit of acute myocardial infarction in Latvia. Int. J. Healthc. Manag..

[20] Lenzi J., Reno C., Skrule J., Lepiksone J., Briģis Ģ., Dūdele A., Fantini M.P. (2020). Excess Cardiovascular Mortality in Latvia: A Novel Approach Based on Patient-Level Data to Estimate the Separate Contributions of Primary Prevention, Accessibility and Quality of Hospital Care. Int. J. Health Policy Manag..

[21] Pandolfi F., Guillemot D., Watier L., Brun-Buisson C. (2022). Trends in bacterial sepsis incidence and mortality in France between 2015 and 2019 based on National Health Data System (Système National des données de Santé (SNDS)): A retrospective observational study. BMJ Open.

[22] Sundararajan V., Henderson T., Perry C., Muggivan A., Quan H., Ghali W.A. (2004). New ICD-10 version of the Charlson comorbidity index predicted in-hospital mortality. J. Clin. Epidemiol..

[23] Charlson M.E., Pompei P., Ales K.L., MacKenzie C.R. (1987). A new method of classifying prognostic comorbidity in longitudinal studies: Development and validation. J. Chronic Dis..

[24] Republic of Latvia Cabinet Regulations. Procedures for the Organisation of and Payment for Health Care Services. Cabinet Regulation. August 2018. https://likumi.lv/ta/en/en/id/301399.

[25] NHS Gada Publiskais Pārskats|Nacionālais Veselības Dienests [In Latvian]. https://www.vmnvd.gov.lv/lv/gada-publiskais-parskats.

[26] NHS Ambulatoro Apmeklējumu Skaits pa Aprūpes Epizodes Veidiem—Datu Kopas—Latvijas Atvērto Datu Portāls [In Latvian]. https://data.gov.lv/dati/lv/dataset/ambulatoro-apmeklejumu-skaits-pa-aprupes-epizodes-veidiem.

[27] Dupuis C., Bouadma L., Ruckly S., Perozziello A., Van-Gysel D., Mageau A., Mourvillier B., de Montmollin E., Bailly S., Papin G. (2020). Sepsis and septic shock in France: Incidences, outcomes and costs of care. Ann. Intensive Care.

[28] Rhee C., Gohil S., Klompas M. (2014). Regulatory Mandates for Sepsis Care—Reasons for Caution. N. Engl. J. Med..

[29] Fleischmann-Struzek C., Thomas-Rüddel D.O., Schettler A., Schwarzkopf D., Stacke A., Seymour C.W., Haas C., Dennler U., Reinhart K. (2018). Comparing the validity of different ICD coding abstraction strategies for sepsis case identification in German claims data. PLoS ONE.

[30] Rhee C., Dantes R., Epstein L., Murphy D.J., Seymour C.W., Iwashyna T.J., Kadri S.S., Angus D.C., Danner R.L., Fiore A.E. (2017). Incidence and Trends of Sepsis in US Hospitals Using Clinical vs Claims Data, 2009-2014. JAMA.

[31] Mellhammar L., Wullt S., Lindberg Å., Lanbeck P., Christensson B., Linder A. (2016). Sepsis Incidence: A Population-Based Study. Open Forum Infect. Dis..

[32] Yébenes J.C., Ruiz-Rodriguez J.C., Ferrer R., Clèries M., Bosch A., Lorencio C., Rodriguez A., Nuvials X., Martin-Loeches I., SOCMIC (Catalonian Critical Care Society) Sepsis Working Group (2017). Epidemiology of sepsis in Catalonia: Analysis of incidence and outcomes in a European setting. Ann. Intensive Care.

[33] Kim J., Kim K., Lee H., Ahn S. (2019). Epidemiology of sepsis in Korea: A population-based study of incidence, mortality, cost and risk factors for death in sepsis. Clin. Exp. Emerg. Med..

[34] Li L., Sunderland N., Rathnayake K., Westbrook J. (2021). Sepsis epidemiology in Australian Public Hospitals, a nationwide longitudinal study (2013-2018). Infect. Dis. Heal..

[35] Imaeda T., Nakada T.-A., Takahashi N., Yamao Y., Nakagawa S., Ogura H., Shime N., Umemura Y., Matsushima A., Fushimi K. (2021). Trends in the incidence and outcome of sepsis using data from a Japanese nationwide medical claims database-the Japan Sepsis Alliance (JaSA) study group-. Crit. Care.

[36] Fleischmann C., Thomas-Rueddel D.O., Hartmann M., Hartog C.S., Welte T., Heublein S., Dennler U., Reinhart K. (2016). Hospital Incidence and Mortality Rates of Sepsis: An Analysis of Hospital Episode (DRG) Statistics in Germany From 2007 to 2013. Dtsch. Aerzteblatt Int..

[37] Fleischmann-Struzek C., Rose N., Freytag A., Spoden M., Prescott H.C., Schettler A., Wedekind L., Ditscheid B., Storch J., Born S. (2021). Epidemiology and Costs of Postsepsis Morbidity, Nursing Care Dependency, and Mortality in Germany, 2013 to 2017. JAMA Netw. Open.

[38] Puceta L., Dumpis U. (2019). Clinical characteristics and outcomes for patients with a sepsis-related diagnosis in multidisciplinary hospital in Latvia. Eur. J. Case Rep. Intern. Med..

[39] Brīdiņa L., Krūmiņa A., Šuba O., Cauce V., Vanags I., Vīksna L. (2016). Severe Sepsis—Clinical Manifestations and Pharmaco-Economic Analysis in an Intensive Care Unit in Latvia. Proc. Latv. Acad. Sci. Sect. B Nat. Exact Appl. Sci..

[40] Davis J.S., He V., Anstey N.M., Condon J.R. (2014). Long Term Outcomes Following Hospital Admission for Sepsis Using Relative Survival Analysis: A Prospective Cohort Study of 1,092 Patients with 5 Year Follow Up. PLoS ONE.

[41] Shankar-Hari M., Harrison D.A., Ferrando-Vivas P., Rubenfeld G.D., Rowan K. (2019). Risk Factors at Index Hospitalization Associated With Longer-term Mortality in Adult Sepsis Survivors. JAMA Netw. Open.

[42] Pandolfi F., Brun-Buisson C., Guillemot D., Watier L. (2022). One-year hospital readmission for recurrent sepsis: Associated risk factors and impact on 1-year mortality—A French nationwide study. Crit. Care.

[43] Wang H.E., Szychowski J.M., Griffin R., Safford M.M., Shapiro N.I., Howard G. (2014). Long-term mortality after community-acquired sepsis: A longitudinal population-based cohort study. BMJ Open.

[44] Spoden M., Hartog C.S., Schlattmann P., Freytag A., Ostermann M., Wedekind L., Storch J., Reinhart K., Günster C., Fleischmann-Struzek C. (2022). Occurrence and Risk Factors for New Dependency on Chronic Care, Respiratory Support, Dialysis and Mortality in the First Year After Sepsis. Front. Med..

[45] Shankar-Hari M., Ambler M., Mahalingasivam V., Jones A., Rowan K., Rubenfeld G.D. (2016). Evidence for a causal link between sepsis and long-term mortality: A systematic review of epidemiologic studies. Crit. Care.

[46] Shankar-Hari M., Saha R., Wilson J., Prescott H.C., Harrison D., Rowan K., Rubenfeld G.D., Adhikari N.K.J. (2020). Rate and risk factors for rehospitalisation in sepsis survivors: Systematic review and meta-analysis. Intensive Care Med..

[47] Schmidt K.F.R., Huelle K., Reinhold T., Prescott H.C., Gehringer R., Hartmann M., Lehmann T., Mueller F., Reinhart K., Schneider N. (2022). Healthcare Utilization and Costs in Sepsis Survivors in Germany–Secondary Analysis of a Prospective Cohort Study. J. Clin. Med..

